# Hidden parasite diversity in a European freshwater system

**DOI:** 10.1038/s41598-020-59548-5

**Published:** 2020-02-14

**Authors:** Christian Selbach, Miroslava Soldánová, Christian K. Feld, Aneta Kostadinova, Bernd Sures

**Affiliations:** 1 Department of Aquatic Ecology, University of Duisburg-Essen, 45141 Essen, Germany; 2 Present Address: Department of Biology, Aquatic Biology, Aarhus University, 8000 Aarhus C, Denmark; 3 Institute of Parasitology, Biology Centre, Czech Academy of Sciences, 37005 České Budějovice, Czech Republic; 4 Institute of Biodiversity and Ecosystem Research, Bulgarian Academy of Sciences, 2 Gagarin Street, 1113 Sofia, Bulgaria; 5 Department of Zoology, University of Johannesburg, Johannesburg, South Africa

**Keywords:** Biodiversity, Ecosystem ecology

## Abstract

Parasites comprise a huge part of the biodiversity on earth. However, on a local scale, not much is known about their diversity and community structure. Here, we assess the diversity of larval trematode communities in an interconnected freshwater system of the River Ruhr in Germany and analyse how the parasites are spatially and temporally distributed in the ecosystem. A total of 5347 snail hosts belonging to six species revealed a highly diverse parasite fauna with 36 trematode species. More abundant snail species harboured more species-rich trematode faunas and communities, with the two dominant snail species, *Radix auricularia* and *Gyraulus albus*, accounting for almost 90% of the trematode diversity and harbouring spatially and temporally stable parasite communities. The results highlight the important role of stable keystone host populations for trematode transmission, structure and diversity. This local trematode diversity reveals information on definitive host occurrence and trophic interactions within ecosystems.

## Introduction

The last 25 to 30 years have seen a slow but steady advancement of our understanding of parasites as integral elements of healthy and functioning ecosystems^1,2^. Nowadays it is considered impossible to fully understand ecosystems without considering parasites^3^. For example, parasites can act as ecosystem engineers^4^ and shape and regulate host population dynamics^5–7^. They often alter predator-prey interactions^8–11^, making them important structuring forces in ecological food webs^12–14^. Moreover, despite their small size, parasites make up a large proportion of an ecosystem’s biomass (e.g.^15–17^.) and thus contribute considerably to the energy flow within ecosystems^18^. In contrast to their typically assumed negative impact, parasites can perform essential functions in an ecosystem, such as concentrating and removing pollutants from the environment^19,20^. Besides contributing to these ecosystem processes, parasites may serve as bioindicators to assess environmental conditions and changes due to their often complex life cycles and strong interactions in ecosystems^21–25^.

There is increasing awareness that parasites are not only important ecosystem components but themselves comprise a huge part of the biodiversity on earth^26^. However, global parasite biodiversity research is far from showing a complete picture, leaving large gaps in our understanding of the diversity and distribution of parasites across ecosystems and hosts^27,28^. The currently highly patchy research effort on parasite diversity not only prevents a full inventory of parasite biodiversity but also impedes predictions of where and when new diseases may emerge^29^. Future environmental changes, such as climate change and other anthropogenic impacts, will have drastic impacts on free-living and parasite communities and ultimately on whole ecosystems^30–32^. However, without a clear understanding of the distribution and structure of parasite communities at local scales we will not be able to measure or even notice these impacts, let alone accurately predict them.

In order to help close this gap, this study focuses on the diversity and distribution of trematode communities in man-made waterbodies, such as impounded lakes and freshwater reservoirs that play a central role in the water management in urban and industrial regions and are common throughout Europe. With a cosmopolitan distribution and about 25,000 species, digenean trematodes are a major parasitic group and constitute the most common eukaryotic pathogens in aquatic ecosystems^33^. These parasites have complex life cycles, involving molluscs as first intermediate hosts and a wide range of invertebrate and vertebrate second intermediate and definitive hosts. The requirement of an obligate, and often species-specific, first intermediate molluscan host, usually a gastropod, makes trematodes ideal model systems to study the distribution and structure of parasite diversity. Furthermore, because trematode communities in snails reflect the richness and abundance of their free-living hosts, they are suitable bioindicators of free-living diversity that allow to assess the complex interactions within ecosystems^34^.

Preliminary studies have revealed that the man-made lakes of the River Ruhr catchment area in Germany offer ideal conditions to study trematode community composition and structure in snails, the first intermediate hosts of the parasites^35,36^. In two consecutive sampling campaigns during the summer months of 2012 and 2013 we have built an extensive dataset on snail-trematode associations from five interconnected lakes of the River Ruhr and its tributaries. The application of integrative morphological and molecular approaches has revealed new and yet unknown species-level lineages in several trematode genera^37–39^. The detailed information on the trematodes gathered so far enables us to provide an accurate overview of the overall diversity of trematode communities in snails and analyse how the parasites are distributed within the interconnected lake system.

The aims of the study are therefore to (i) assess the diversity of digenean trematodes in lymnaeid and planorbid snails in five lakes of the Ruhr River system; (ii) analyse temporal and spatial variation in parasite component community structure and composition; and (iii) based on the knowledge of the parasites’ life cycles, identify transmission pathways that can reveal information on definitive host occurrence and trophic interactions in the ecosystems. In order to achieve these aims, we performed extensive sampling of trematode communities in snails in the River Ruhr and identified parasite transmission pathways in the system studied, based on published life cycle data.

## Results

### Overall trematode prevalence

A total of 5347 snails (3171 lymnaeids of four species and 2176 planorbids of two species), was sampled and analysed for infections with digenean trematodes during 2012 and 2013. Distribution and abundance of snail populations varied between lakes, with the majority of planorbid snails being found at Hennetalsperre and most lymnaeid species at the remaining lakes (Table 1). Of the 5347 snails, 1049 harboured patent or prepatent trematode infections, resulting in an overall prevalence of 19.6%. The overall prevalence was highly variable among the different lakes and snail species, ranging from 2.6% in *Segmentina nitida* to 31.7% in *Radix auricularia* (Table 1).

**Table 1:** Total numbers of the six snail species sampled in the five Ruhr lakes in 2012 and 2013, with the number of examined snails, infected snails, overall prevalence of trematode infections (% of infected snails in the pooled samples), and number of samples used in the component community analyses.

Lake		Baldeneysee			Hengsteysee			Sorpetalsperre			Hennetalsperre			Versetalsperre			Total
Year		2012	2013	Total	2012	2013	Total	2012	2013	Total	2012	2013	Total	2012	2013	Total	
**Lymnaeidae**																	
***Radix auricularia***	No. examined	275	91	**366**	596	353	**949**	248	90	**338**	220	36	**256**	—	—	—	**1909**
	No. infected	46	15	**61**	178	132	**310**	99	56	**155**	70	10	**80**	—	—	—	**606**
	Prevalence	16.7	16.5	**16.7**	29.9	37.4	**32.7**	39.9	62.2	**45.9**	31.8	27.8	**31.3**	—	—	—	**31.7**
	No. of samples^a^	10 (8)	3 (3)	**13 (11)**	12 (11)	11 (9)	**23 (20)**	9 (9)	3 (3)	**12 (12)**	5 (5)	0	**5 (5)**	—	—	—	**53 (48)**
***Radix peregra***	No. examined	—	—	—	—	—	—	—	—	—	16	20	**36**	294	19	**313**	**349**
	No. infected	—	—	—	—	—	—	—	—	—	12	7	**19**	7	1	**8**	**27**
	Prevalence	—	—	—	—	—	—	—	—	—	75.0	35.0	**52.8**	2.4	5.5	**2.6**	**7.7**
	No. of samples^a^	—	—	—	—	—	—	—	—	—	1 (1)	0	**1 (1)**	4 (3)	0	**4 (3)**	**5 (4)**
***Lymnaea stagnalis***	No. examined	28	8	**36**	100	108	**208**	—	1	**1**	—	—	—	—	—	—	**245**
	No. infected	5	2	**7**	12	21	**33**	—	0	**0**	—	—	—	—	—	—	**40**
	Prevalence	17.9	25.0^b^	**19.4**	12.0	19.4	**15.9**	—	0	**0**	—	—	—	—	—	—	**16.3**
	No. of samples^a^	0	0	**0**	2 (2)	4 (4)	**6 (6)**	—	0	**0**	—	—	—	—	—	—	**6 (6)**
***Stagnicola palustris***	No. examined	29	6	**35**	530	85	**615**	15	3	**18**	—	—	—	—	—	—	**668**
	No. infected	3	3	**6**	75	6	**81**	1	1	**2**	—	—	—	—	—	—	**89**
	Prevalence	10.3	50.0^b^	**17.1**	14.5	7.1	**13.2**	6.7	33.3^b^	**11.1**	—	—	—	—	—	—	**13.3**
	No. of samples^a^	0	0	**0**	7 (6)	2 (1)	**9 (7)**	0	0	**0**	—	—	—	—	—	—	**9 (7)**
**Planorbidae**																	
***Gyraulus albus***	No. examined	5	14	**19**	2	26	**28**	—	—	—	1,098	830	**1928**	1	5	**6**	**1981**
	No. infected	0	8	**8**	0	2	**2**	—	—	—	157	114	**271**	1	0	**1**	**282**
	Prevalence	0	57.1	**42.1**	0	7.7	**7.1**	—	—	—	14.3	13.7	**14.1**	100.0^b^	0	**16.7**^**b**^	**14.2**
	No. of samples^a^	0	0	**0**	0	1 (0)	**1 (0)**	—	—	—	6 (6)	5 (4)	**11 (10)**	0	0	**0**	**12 (11)**
***Segmentina nitida***	No. examined	15	—	**15**	—	—	—	—	—	—	127	53	**180**	—	—	—	**195**
	No. infected	0	—	**0**	—	—	—	—	—	—	2	3	**5**	—	—	—	**5**
	Prevalence	0	—	**0**	—	—	—	—	—	—	1.6	5.7	**2.8**	—	—	—	**2.6**
	No. of samples^a^	0	—	**0**	—	—	—	—	—	—	4 (1)	1 (1)	**5 (2)**	—	—	—	**5 (2)**

^a^Only samples consisting of n ≥ 14 snails were used in component community analyses; numbers in parentheses show samples with trematode infections. ^b^Sample size small (n < 14), excluded from component community analyses.

### Composition and diversity of the trematode faunas

**Figure 1:**
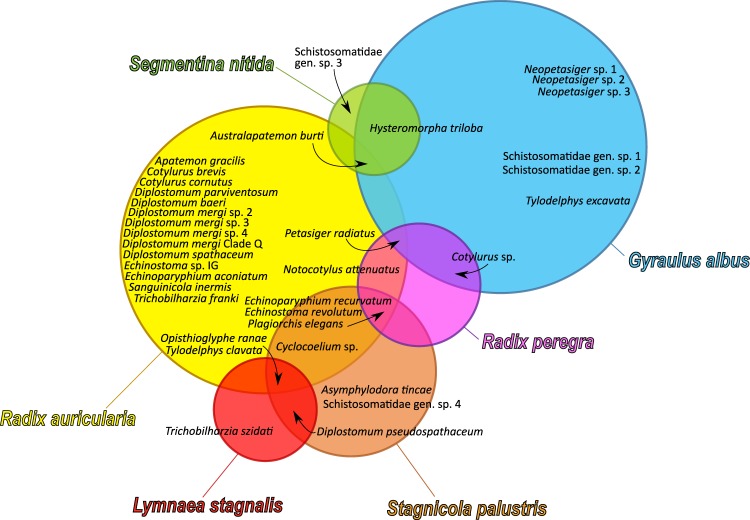
Graphical representation of the snail species and their trematode fauna in the Ruhr lakes. Each circle represents one host snail species, with the area of each circle corresponding to the total number of snails sampled during the study. Trematode species of a particular host are indicated in the respective circles. Areas where circles overlap indicate shared trematode species; arrows show the position of individual species where space was limited.

A total of 36 trematode species belonging to nine families were found. Table 2 shows the overall prevalence of larval trematodes in the six snail hosts per lake. Trematode species richness varied considerably between the snail hosts ranging from three species in *S. nitida* to 23 species in *R. auricularia*. In total, 86% of the larval trematode diversity (31 out of 36 species) was harboured by *R. auricularia* and *G. albus*, the most commonly encountered snail species during our sampling trips (Fig. 1). Overall, the trematode species found in the snail populations utilise a wide variety of second intermediate and definitive vertebrate hosts. However, the majority of trematodes fall within two transmission guilds, either the guild of parasites of fish-eating birds that use fishes as second intermediate and fish-eating birds as definitive hosts (15 species), or the guild of waterfowl parasites that utilise anseriform birds as definitive hosts (15 species). The remaining species utilise as definitive hosts birds of the families Rallidae (one species), Ciconiidae (one species), or amphibians (one species), cyprinids (two species) or mammals and passeriform birds (one species) (Table 2).

**Table 2:** Overall prevalence of the trematode species (% of infected snails in the pooled samples from 2012 and 2013) infecting the six snail species in the River Ruhr.

Snail species	Trematode family	Trematode species	2^nd^ intermediate host	Definitive host	Lake					
					Baldeneysee	Hengsteysee	Sorpetalsperre	Hennetalsperre	Versetalsperre	TOTAL
*Radix auricularia*	Cyclocoelidae	*Cyclocoelum* sp.*	Molluscs (first intermediate host acts as second intermediate host)	Rallid birds	0.3	4.1	0.9	—	—	2.3
	Diplostomidae	*Diplostomum baeri*	Fishes	Fish-eating birds	—	0.1	—	—	—	0.1
		*Diplostomum* sp. “Clade Q”	Fishes	Fish-eating birds	—	0.1	—	—	—	0.1
		*Diplostomum mergi* 2*†	Fishes	Fish-eating birds	0.3	0.7	1.2	—	—	0.6
		*Diplostomum mergi* 3†	Fishes	Fish-eating birds	—	0.4	—	—	—	0.2
		*Diplostomum mergi* 4	Fishes	Fish-eating birds	—	0.1	—	—	—	0.1
		*Diplostomum parviventosum*	Fishes	Fish-eating birds	—	1.0	—	—	—	0.5
		*Diplostomum spathaceum*†	Fishes	Fish-eating birds	—	0.7	—	—	—	0.4
		Tylodelphys clavata*†	Fishes	Fish-eating birds	1.1	1.2	1.8	3.1		1.5
	Echinostomatidae	Echinoparyphium aconiatum*†	Molluscs	Waterfowl	—	0.2	0.3	3.5	—	0.6
		Echinoparyphium recurvatum*†	Molluscs	Waterfowl	0.3	1.5	13.0	3.9	—	3.6
		Echinostoma revolutum*	Molluscs	Waterfowl	—	—	—	2.3	—	0.3
		*Echinostoma* sp. IG	Molluscs	Waterfowl	0.3	—	0.3	—	—	0.1
		Petasiger radiatus* †^a^	Fishes	Fish-eating birds	12.0	9.9	11.5	6.6	—	10.2
	Notocotylidae	Notocotylus attenuatus*†	None (cercariae encyst on vegetation)	Waterfowl	0.8	2.2	4.4	4.7	—	2.7
	Plagiorchiidae	Plagiorchis elegans*	Larval insects, amphipods, molluscs	Various birds, mammals	—	7.0	4.7	4.7	—	4.9
	Sanguinicolidae	*Sanguinicola inermis*	None (direct life cycle)	Cyprinids	—	—	1.2	—	—	0.2
	Schistosomatidae	Trichobilharzia franki*†	None (direct life cycle)	Waterfowl	0.6	0.4	5.0	0.4	—	1.3
	Strigeidae	*Apatemon gracilis*	Fishes	Fish-eating birds	—	0.11	—	—	—	0.1
		*Australapatemon burti* †	Leeches	Waterfowl	0.27	1.0	0.9	—	—	0.7
		*Cotylurus brevis*	Molluscs, leeches	Waterfowl	—	—	—	0.8	—	0.1
		Cotylurus cornutus*†	Molluscs, leeches	Waterfowl	—	0.6	1.2	—	—	0.5
	Telorchiidae	*Opisthioglyphe ranae*	Amphibians	Amphibians	0.8	0.3	—	0.4	—	0.4
*Radix peregra*	Echinostomatidae	Echinoparyphium recurvatum*	Molluscs	Waterfowl	—	—	—	13.9	1.6	2.9
		Echinostoma revolutum*	Molluscs	Waterfowl	—	—	—	16.7	—	1.7
		*Petasiger radiatus*^a^	Fishes	Fish-eating birds	—	—	—	11.1	—	1.2
	Notocotylidae	*Notocotylus attenuatus*	None (cercariae encyst on vegetation)	Waterfowl	—	—	—	2.8	—	0.3
	Plagiorchiidae	*Plagiorchis elegans*	Molluscs, larval insects, crustaceans	Various birds, mammals	—	—	—	2.8	0.6	0.9
	Strigeidae	*Cotylurus* sp.	Molluscs, leeches	Waterfowl	—	—	—	2.8	—	0.3
*Lymnaea stagnalis*	Diplostomidae	Diplostomum pseudospathaceum*	Fishes	Fish-eating birds	13.9	10.6	—	—	—	11.0
		*Tylodelphys clavata*	Fishes	Fish-eating birds	5.6		—	—	—	0.8
	Schistosomatidae	*Trichobilharzia szidati*	None (direct life cycle)	Waterfowl		1.0	—	—	—	0.8
	Telorchiidae	*Opisthioglyphe ranae*	Amphibians	Amphibians	2.8	1.4	—	—	—	1.6
*Stagnicola palustris*	Cyclocoelidae	*Cyclocoelum* sp.*	Molluscs (first intermediate host acts as second intermediate host)	Rallid birds	—	8.9	—	—	—	8.2
	Diplostomidae	Diplostomum pseudospathaceum*	Fishes	Fish-eating birds	5.7	2.6	—	—	—	2.7
		*Tylodelphys clavata*	Fishes	Fish-eating birds	2.9	0.2	—	—	—	0.3
	Echinostomatidae	*Echinoparyphium recurvatum*	Molluscs	Waterfowl	—	0.2	—	—	—	0.2
		*Echinostoma revolutum*	Molluscs	Waterfowl	—	0.2	—	—	—	0.2
	Lissorchiidae	*Asymphylodora tincae*	None	Cyprinids	—	0.2	—	—	—	0.2
	Plagiorchiidae	*Plagiorchis elegans*	Molluscs, larval insects, crustaceans	Various birds, mammals	—	0.3	5.6	—	—	0.5
	Schistosomatidae	Schistosomatidae gen. sp. 4	None (direct life cycle)	Waterfowl	—	0.2	—	—	—	0.2
	Telorchiidae	*Opisthioglyphe ranae*	Amphibians	Amphibians	8.6	0.3	—	—	—	0.8
*Gyraulus albus*	Diplostomidae	*Hysteromorpha triloba*	Fishes	Fish-eating birds	—	3.6	—	3.3	—	3.3
		*Tylodelphys excavata*	Amphibians	Storks	5.3	—	—	—	—	0.1
	Echinostomatidae	*Neopetasiger* sp. 1^b^	Fishes	Fish-eating birds	—	—	—	0.2	—	0.2
		*Neopetasiger* sp. 2^b^	Fishes	Fish-eating birds	—	—	—	0.1	—	0.1
		*Neopetasiger* sp. 3^b^	Fishes	Fish-eating birds	—	3.6	—	0.1	—	0.2
		*Petasiger radiatus*^a^	Fishes	Fish-eating birds	26.3	—	—	2.2	—	2.4
	Schistosomatidae	Schistosomatidae gen. sp. 1	None (direct life cycle)	Waterfowl	—	—	—	0.1	—	0.1
		Schistosomatidae gen. sp. 2	None (direct life cycle)	Waterfowl	—	—	—	0.2	—	0.2
	Strigeidae	Australapatemon burti*	Leeches	Waterfowl	—	—	—	7.5	16.7 ^c^	7.3
		*Cotylurus* sp.	Molluscs, leeches	Waterfowl	10.5	—	—	—	—	0.1
*Segmentina nitida*	Diplostomidae	*Hysteromorpha triloba*	Fishes	Fish-eating birds	—	—	—	0.6	—	0.5
	Schistosomatidae	Schistosomatidae gen. sp. 3	None (direct life cycle)	Waterfowl	—	—	—	0.6	—	0.5
	Strigeidae	*Australapatemon burti*	Leeches	Waterfowl	—	—	—	1.1	—	1.0

Lakes where prevalence was zero or no suitable host populations were found are indicated by a minus (−). Intermediate and definitive hosts are provided based on literature data^58,59,62,68–72^.

^a^Syn. *Paryphostomum radiatum*^73^.

^b^Described as *Petasiger* spp. 1–3 by Selbach *et al*.^38^.

^c^Sample size small (n < 14).

*Dominant species, i.e. with a prevalence ≥ 10% in at least one component community.

^†^Most common species in the guilds of waterfowl and fish-eating bird parasites that were used in the ANCOVAs.

### Component community structure

**Figure 2:**
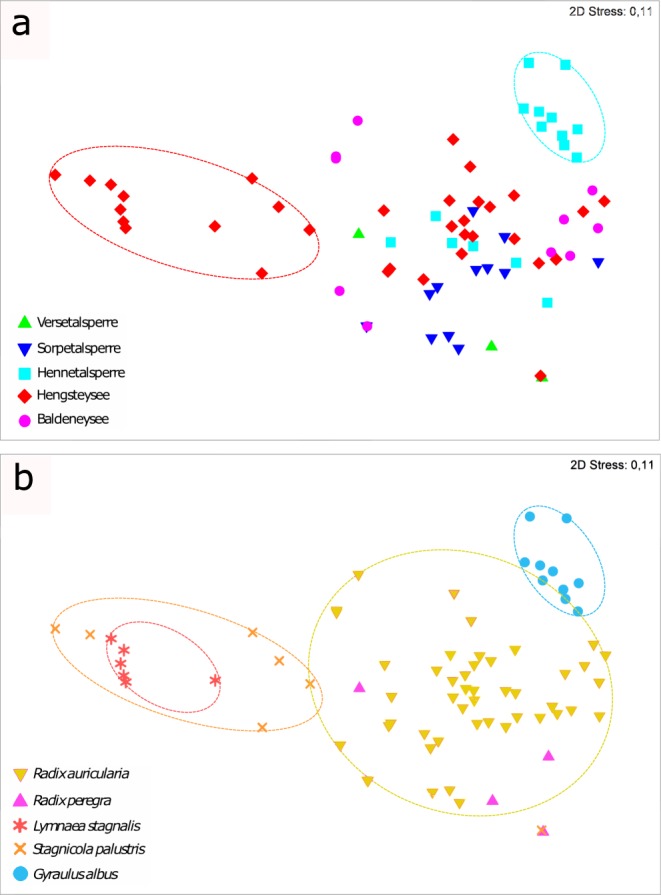
Two-dimensional MDS ordination plots of 75 trematode component communities based on the similarity in trematode component community structure (Bray-Curtis index, stress value = 0.11). (**a**) Ordination plot with indication for lake. (**b**) Ordination plot with indication for snail host, with ellipses drawn to highlight the communities in the different snail hosts.

For the analysis of trematode component communities, only samples comprising more than 14 snails were used (see Methods), resulting in a total of 75 community samples (48 samples for *R. auricularia*, 10 for *G. albus*, seven for *S. palustris*, six for *L. stagnalis* and four for *R. peregra*). The MDS ordination plot showed no clear overall pattern for the factor ‘lake,’ except for two distinctive clusters in Hennetalsperre and Hengsteysee (Fig. 2a). Accordingly, although the ANOSIM test revealed a significant difference in composition and structure of trematode component communities for the factor ‘lake’, the low R-statistic indicates little differentiation between the water bodies (R = 0.163, p = 0.001). These distinctive groups are evidently due to the different snail host populations of these lakes, as the plot for the factor ‘snail’ reveals (Fig. 2b). The plot highlights three distinctive groups and a good separation between: (i) communities in *Lymnaea stagnalis* and *Stagnicola palustris* from Hengsteysee; and (ii) communities in *Gyraulus albus* from Hennetalsperre, and *Radix* spp. from all lakes. This is supported by the ANOSIM test (R = 0.567; p = 0.001) indicating some overlap (*R. auricularia* and *R. peregra* in particular) but also a clear separation for the factor ‘snail host’, revealing dissimilarities in trematode communities in the five snail hosts. No effects for the factors ‘season’ and ‘year’ were detected by one-way ANOSIM analyses (all p > 0.05). However, the artefactual effects of the three different host groups highlighted in the MDS might distort these results. For this reason, parasite communities in *Radix auricularia* were analysed for temporal and spatial variation in detail (see below).

### Temporal and spatial variation of trematode communities in *Radix auricularia*

Sample size differed significantly between lakes (ANOVA; F_2, 40_ = 5.01, p = 0.01), particularly between Baldeneysee and Hengsteysee with larger samples obtained in the latter (Table 1). In contrast, sample sizes did not differ between seasons and years (p > 0.05). Snail size (mean shell length) differed between lakes (ANOVA; F_2, 40_ = 37.15, p < 0.001) with snails being generally larger in Sorpetalsperre (mean length ± SD, 19.06 ± 2.38 *vs* 11.83 ± 2.22 mm in Baldeneysee and 13.41 ± 1.78 mm in Hengsteysee). Whereas differences were detected between seasons (ANOVA; F_2, 45_ = 5.32, p < 0.01), with larger snails in the spring samples, snails of similar sizes were generally found in summer and autumn and in both years (p > 0.05). There was no significant correlation between sample size and trematode species richness (r_s,_ = 0.83, p > 0.05). However, mean snail size was positively correlated with overall prevalence (r_s_ = 0.41, p < 0.01) and with species richness (r_s_ = 0.31, p < 0.05), revealing higher infection levels and more parasite species in populations with larger, i.e. older snails.

In order to avoid confounding effects of snail size while evaluating possible differences in overall prevalence between lakes, seasons and years, we performed sets of ANCOVAs using ‘season’, ‘lake’ and ‘year’ as factors and ‘mean snail length’ as a covariate. After the effect of snail size was accounted for, no significant differences in overall prevalence were detected between lakes, seasons and years in both separate two-way ANCOVAs (Table 3; ANCOVA 1 carried out on 43 samples from three lakes and three seasons and ANCOVA 2 carried out on 37 samples from three lakes and two seasons, i.e. summer and autumn, see Methods). Although relatively higher overall prevalence was observed at Sorpetalsperre (Table 1), it was not significantly higher than in other lakes, indicating spatially and temporarily stable communities in *R. auricularia* in the Ruhr River ecosystem.

**Table 3:** ANCOVA statistics for the variation in overall trematode prevalence in *Radix auricularia* and the prevalence of infection of the two major transmission guilds as a function of lake, season and year and their interactions.

Variable	Factor	ANCOVA 1			ANCOVA 2		
		3 lakes and 3 seasons			3 lakes and 2 seasons		
		df	F	P	df	F	P
Overall prevalence	Lake	2, 33	1.51	ns	2, 30	1.80	ns
	Season	2, 33	0.79	ns	1, 30	0.03	ns
	Lake and season	4, 33	0.36	ns	2, 30	0.55	ns
	Lake	2, 36	2.57	ns	2, 30	1.99	ns
	Year	1, 36	2.69	ns	1, 30	1.21	ns
	Lake and year	2, 36	1.58	ns	2, 30	0.98	ns
	Season	—	—	—	1, 32	0.16	ns
	Year	—	—	—	1, 32	2.31	ns
	Season and year	—	—	—	1, 32	1.96	ns
Waterfowl guild prevalence	Lake	2, 33	4.06	<0.05	2, 30	6.13	<0.01
	Season	2, 33	0.36	ns	1, 30	0.53	ns
	Lake and season	4, 33	3.19	<0.05	2, 30	2.76	ns
	Lake	2, 36	6.61	<0.01	2, 30	12.36	<0.001
	Year	1, 36	2.58	ns	1, 30	3.76	ns
	Lake and year	2, 36	0.26	ns	2, 30	1.85	ns
	Season	—	—	—	1, 32	0.07	ns
	Year	—	—	—	1, 32	4.23	0.048*
	Season and year	—	—	—	1, 32	0.27	ns
Fish-eating bird guild prevalence	Lake	2, 33	0.69	ns	2, 30	0.81	ns
	Season	2, 33	1.96	ns	1, 30	0.29	ns
	Lake and season	4, 33	1.88	ns	2, 30	3.39	0.047*
	Lake	2, 36	0.35	ns	2, 30	0.06	ns
	Year	1, 36	5.34	<0.05	1, 30	2.97	ns
	Lake and year	2, 36	1.41	ns	2, 30	1.38	ns
	Season	—	—	—	1, 32	1.13	ns
	Year	—	—	—	1, 32	7.22	<0.05
	Season and year	—	—	—	1, 32	3.42	ns

^*^No differences detected in post-hoc Tukey’s test.

*Abbreviation*: ns = not significant.

On the other hand, detailed analyses carried out on the prevalence of two major transmission guilds revealed a significant variation between lakes for trematodes utilising waterfowl as definitive hosts, and significant differences in prevalence between years for trematodes using fish-eating birds (Table 3). However, these differences were not pronounced enough to affect the spatio-temporal overall prevalence pattern (see above).

There were no significant differences among seasons for the prevalence of both guilds (p > 0.05; Table 3). The detected differences among lakes for the waterfowl guild parasites are likely due to the high prevalence of these groups in Sorpetalsperre compared to Hengsteysee and Baldeneysee (ANCOVA 1 dataset: 25.2% *vs* 6.6% and 1.6% respectively; ANCOVA 2 data set: 30.2% *vs* 6.5% and 1.3%). The prevalence of the waterfowl guild parasites at Hengsteysee in both summer (5.7%) and autumn (9.4%) was considerably lower than in summer at Sorpetalsperre (32.5%). Furthermore, there was a low prevalence in summer communities at Baldeneysee (0.9%) compared to spring and summer at Sorpetalsperre (14.1% and 32.5%, respectively). This resulted in significant interaction between ‘lake’ and ‘season’ in the analysis ANCOVA 1, but no such effect was detected in the restricted data set (ANCOVA 2). Although summer prevalence at Baldeneysee remained different from summer at Sorpetalsperre (ANCOVA 1), autumn samples differed too (23.6%, Sorpetalsperre). This is because spring samples were excluded from ANCOVA 2 and there was no effect which would lead to interactions between factors ‘lake’ and ‘season’ (Table 3). These patterns are consistent with prevalence of the most dominant species in the waterfowl guild, i.e., *Echinoparyphium recurvatum* and *Notocotylus attenuatus*.

The prevalence of trematodes of fish-eating birds differed significantly between years in both data sets, with lower levels in 2012 in comparison to 2013 (10.5% *vs* 24.3%; ANCOVA1). This effect was also apparent using the restricted dataset with no spring samples (11.7% in 2012 *vs* 24.4% in 2013; ANCOVA 2, Table 3). Although, there was an interaction between ‘lake’ and ‘season’ in the ANCOVA 2 dataset, no differences were detected by post-hoc Tukey’s tests (Table 3). The above patterns for the fish-eating bird guild are consistent with the prevalence of the one most dominant species in this guild, i.e. *Petasiger radiatus*, which exhibited generally high prevalence in 2013 (21.1% *vs* 7.7% in 2012) compared to other species in this guild (range of 0.4–1.8% in 2013 *vs* 0.5–1.6% 2012).

## Discussion

This is the first large-scale study of trematode diversity in an interconnected freshwater system. Altogether, the 5347 pulmonate snails belonging to six species revealed a species-rich and diverse trematode fauna in the Ruhr lakes with a total of 36 trematode species belonging to nine families. This is considerably higher than the trematode species richness in snail intermediate hosts usually described from most other well-studied freshwater ecosystems (see Table 4). The most comparable study comes from a survey in Western Canada, collecting five snail species from six lakes over the course of two years and reporting 39 trematode species identified with the aid of morphological and molecular tools^40^. This assessment had a much larger sample size (13179 snails) and included three snail host families (Lymnaeidae, Planorbidae and Physidae), but both study design and findings are highly similar, highlighting the high diversity of trematode communities in large freshwater bodies in North America and Europe.

**Table 4:** Overview of comparable snail-trematode diversity and community studies.

No. of snail species	No. of snails sampled	No. of. trematode species	Overall prevalence	Sampling sites	Reference
11	14000	18	n/a	Estuarine saltmarsh system in the U.S.	^15^
1	n/a	11	‘low’	Four lakes in New Zealand	^74^
5	13197	39	13.5%	Six lakes in Canada	^40^
1	10821	6 genera	8.8%	120 freshwater ponds in the U.S.	^75^
15	6403	29	4.9%	Pooled data from rivers, ponds and lakes in Germany	^42^
12	2802	26	33.9%	Two fishponds and one swamp in the Czech Republic	^43^
6	10581	25	46.5%	29 lakes in Poland	^44^
14	n/a	26	n/a	Seven lakes in Poland	^76^
**6**	**5347**	**36**	**19.6%**	**Interconnected Ruhr lake system**	**Present study**

Among the snail species studied by us, *Radix auricularia* harboured the most species-rich and diverse trematode fauna of all studied hosts in the Ruhr River system (23 species), and by far outnumbers the 12 trematode species previously found in this host in the Ruhr River^35^. The five cryptic species revealed by molecular methods in the genera *Diplostomum* and *Echinostoma*^37,39^ contributed to this high diversity. Overall, this high species richness is in stark contrast to what is described from the literature. Initial compilations of the cercariae species recorded in lymnaeid snails counted six species in *R. auricularia* (^41^ and references therein), and sampling data from Southeast Germany (four trematode species^42^), the Czech Republic (three species^43^) and Poland (one species^44^) supported the low number of trematode species in this host. The highest species richness recorded is eleven species of cercariae recovered from *R. auricularia* in a gravel pit in the United Kingdom^45^.

The remarkably high trematode species richness in *R. auricularia* in the Ruhr system supports the assumption that *R. auricularia* constitutes the most important snail host in the life cycle of these parasites in large lakes, in contrast to the more dominant role of *Lymnaea stagnalis* in small pond systems (see^35^ and reference therein), for which 41 trematode species are described from Europe (^46^ and references therein). The only snail species that was equally abundant in our sampling was *G. albus*, but trematode richness and diversity were lower than in *R. auricularia*. In total, 86% of the larval trematode diversity (31 out of 36 species) was harboured by *R. auricularia* and *G. albus*, making these two snail species the most important first intermediate hosts for digenean trematodes in the large interconnected lake system of the Ruhr River.

After the confounding effect of snail size was accounted for, no significant differences in overall prevalence was detected between lakes, seasons and years, indicating spatially and temporarily stable trematode communities in *R. auricularia*. Although the significant difference between trematode component communities was revealed in different lakes, these differences are rather due to the uneven distribution of snail hosts across the waterbodies. Accordingly, this illustrates a clear structure in the trematode communities that indicate distinctive groups of trematode communities in the different snail species, similar to other host-parasite systems^47^. It is, therefore, the composition of snail host populations that shapes the trematode community structure in lakes of the River Ruhr.

Almost all of the trematode species found in *R. auricularia* utilise birds as definitive hosts, mainly either waterfowl or fish-eating birds that overwinter in the Ruhr area, due to the abundant food resources available at the lakes. The large-bodied Ruhr lakes do not fall dry during the summer or freeze over in winter and provide stable conditions for trematode life cycles all year. This continuous presence of definitive bird hosts that can migrate among the lakes and the presence of abundant snail host populations may explain the seasonal and spatial homogeneity in this system. Unlike *L. stagnalis*, *R. auricularia* are relatively short-lived (one to two years^48^), indicating, that trematode populations are quickly re-established every year in the Ruhr River lakes. From the parasites’ perspective, the different snail populations represent habitable islands^49^, and within the Ruhr lakes it is the constant availability of these hosts in the ecosystem that determines the distribution, composition and structure of trematode communities.

Comparable to the findings of Gordy *et al*.^40^, our results suggest that it is not snail host diversity *per se* that determines parasite diversity, but rather the abundance and composition of these host populations. *Radix auricularia* and *G. albus* were equally abundant in our assessment (1909 and 1981 snails, respectively), and harboured the most species-rich trematode faunas. However, the trematodes infecting *R. auricularia* showed a much higher overall species richness and prevalence than those infecting *G. albus*, highlighting the different roles of different host species for a diverse trematode fauna. Consequently, *R. auricularia* can be regarded as a keystone host species^50^ that hosts a disproportionate number of parasite species and boosts the trematode diversity in the Ruhr River system. This finding corresponds with the patterns in marine snail-trematode systems that consider trematode richness and prevalence as traits of individual snail species^51^.

The majority of the 36 trematode species identified in the six snail intermediate host species use birds as definitive hosts to complete their life cycle, only two species require cyprinids (*S. inermis* and *A. tincae*) and one species uses amphibians as definitive hosts (*O. ranae*). Most of the species utilising birds either fall within the large guild of parasites that use fish and fish-eating birds (mainly cormorants, gulls or grebes), or belong to the generalist guild parasitizing waterfowl (anseriform birds) as definitive hosts. The remaining species use either birds of the families Rallidae (rails) or Ciconiidae (storks), or a wider host spectrum of various birds or mammals.

**Figure 3:**
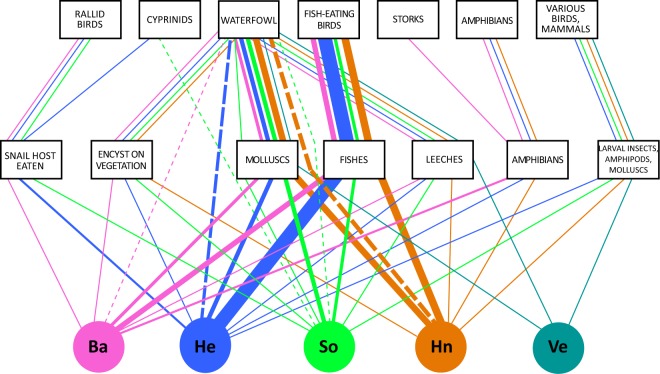
Scheme of transmission pathways of the trematode species found in the snail populations in the five studied lakes. Boxes in the middle represent second intermediate hosts or encystment in the aquatic environment and boxes at the top represent definitive host groups. The lines indicate trematode species utilising individual transmission pathways, with the thickness of the lines being proportional to the number of trematode species utilising each transmission pathway. Dotted lines show direct infection of definitive host. *Abbreviations*: Ba = Baldeneysee; He = Hengsteysee; So = Sorpetalsperre; Hn = Hennetalsperre; Ve = Versetalsperre.

Altogether, this information on the life cycle and required host species allows the reconstruction of the transmission pathways the individual parasite species take through the ecosystem (see Fig. 3). Since the infections of snails require definitive hosts to release eggs into the ecosystem, the transmission pathways provide accurate information on the presence of these hosts in the ecosystem. Especially in the case of highly mobile birds that can migrate long distances and may not be present at the sampling site at all times, the occurrence of trematode infections in snails can provide evidence of the local host distribution. This can be especially useful in the case of rare or migratory species, such as storks in Europe, where trematode infections in snails may be used to map host distribution and movement. Such examples highlight practical applications where trematodes may serve as bioindicators of free-living diversity and species distribution^34^ Definitive hosts are the upstream hosts of trematode infections in snails (see^52^), i.e., snails have become infected via a parasitic stage released by an infected definitive host and we can infer the (at least temporary) presence of this definitive host in the system. On the other hand, we cannot be absolutely certain that the downstream host from the snail, i.e., a suitable second intermediate host for the parasite, is present in the system. However, the continuous presence of most trematode species over two consecutive years, suggest that the life cycles of these parasites can be completed within these lakes. Overall, the stable trematode communities indicate stable intermediate and definitive host presence at the Ruhr River system.

Fishes act as second intermediate hosts for a wide range of taxa but seem to play no role as definitive hosts for the life cycles of trematodes in our study system since just two species (*Asymphylodora tincae* and *Sanguinicola inermis*) were recorded in *S. palustris* and *R. auricularia*, respectively. However, this is not unexpected since 15 out of the 20 trematode species reported in Europe for which fishes act as definitive hosts^46^ utilise either bivalves (three species of the Allocreadiidae; two species of the Azygiidae; three species of the Bucephalidae; and three species of the Gorgoderidae) or different snail hosts (four species of the Opecoelidae using *Bithynia* spp., *Lithoglyphus* spp. or *Theodoxus* spp.) as first intermediate hosts. Thus, the two species recorded in Ruhr lakes represent a third of the known trematode diversity of fish trematodes in Europe for the snail hosts studied.

All transmission strategies, except for the direct infection of definitive hosts by the cercariae (e.g., in bird schistosomes), involve trophic transmission to the definitive host and thus provide information on trophic interactions and energy flow within the ecosystem. This connectivity exposes to what extent intra-host stages of digenean trematodes are embedded in larger food webs. In our case, the large number of transmission events from aquatic organisms to birds, indicate a substantial energy flow from freshwater to terrestrial systems via these predation events. Moreover, trematode infections can have drastic effects on second intermediate hosts and lead to behavioural changes that make parasitized prey easier for predators to capture^8^. Therefore, more than mere ‘blind passengers’, parasites often directly or indirectly manipulate their hosts^7,8,52^ and thereby actively shape the structure of food webs through which they are transmitted, thus regulating host population dynamics and influencing the community structure of free-living species^53^. In our study system, trematodes of the family Diplostomidae were prevalent at several sampling sites. These trematodes parasitize the eyes of their intermediate fish hosts where they impact visual perception and negatively influence the fishes’ feeding behaviour as well as reducing their chances of evading avian predators that constitute the parasites’ definitive hosts^54–56^. Overall, the large percentage of trophically-transmitted parasite species underlines their potential structuring role in food webs of the Ruhr lake ecosystem.

In summary, our study highlights the diversity of trematodes in Ruhr lakes and reveals how trematode communities are distributed within the interconnected freshwater system. Lymnaeid and planorbid snail populations offer suitable habitats for a species rich and abundant trematode fauna, with the two most dominant species, *R. auricularia* and *G. albus*, harbouring almost 90% of the ecosystem’s trematode diversity. Trematode component communities in *R. auricularia* were spatially and temporally stable, indicating stable conditions and the continuous presence of the main trematode hosts in the system. In addition, the diverse communities of trematodes with different life cycle strategies can reveal important information on definitive host occurrence and trophic interactions in the ecosystems.

On the surface, parasites are usually not directly visible in an ecosystem, while their hosts are naturally regarded to constitute the biota that inhabit an ecosystem. For this reason, parasites have traditionally been omitted from the majority of ecological studies^57^. This is deceptive however, since free-living organisms only constitute a small fraction of the whole ecosystem. Beneath the surface, parasites are deeply embedded in and active elements of the ecological processes that shape and structure ecological communities, energy flow and the biodiversity of complex ecosystems. Recognising the diversity and distribution of these organisms, both at global and regional scales, is therefore central to our understanding of ecosystems and their future changes.

## Methods

### Sampling

**Figure 4:**
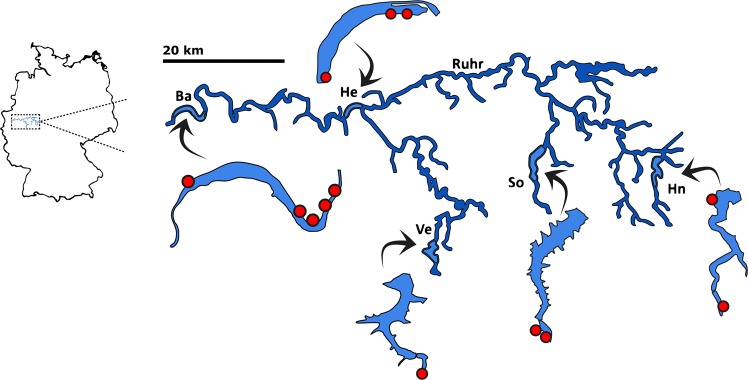
Map of the Ruhr area and the lake system studied. Individual sampling sites are highlighted by red dots. *Abbreviations*: Ba = Baldeneysee; He = Hengsteysee; So = Sorpetalsperre; Hn = Hennetalsperre; Ve = Versetalsperre.

Snail hosts were collected at several sampling sites in five artificial lakes of the Ruhr River catchment area, Germany: Baldeneysee (51°24′20.08″N, 7°2′22.47″E), Hengsteysee (51°24′52.17″N, 7°27′42.55″E), Hennetalsperre (51°19′50.97″N, 8°15′46.82″E), Sorpetalsperre (51°20′15.01″N, 7°56′46.18″E) and Versetalsperre (51°10′55.71″N, 7°40′57.12″E) (Fig. 4). All water bodies were constructed during the first half of the 20th century along the River Ruhr and its tributaries as drinking water reservoirs, natural river water treatment plants and to regulate the water flow of the river system.

At each lake suitable sampling sites were identified and selected based on the following criteria: (i) accessibility; (ii) the presence of aquatic vegetation providing food and shelter for molluscs; and (iii) the presence of potential definitive hosts. Each sampling site was visited repeatedly during 11 sampling campaigns in spring, summer and autumn (May–September) in two consecutive years, 2012 and 2013. Snails were collected by hand or with hand-nets from stones, sediment and aquatic vegetation along selected transect near the shore of each pond (40–45 m long by 1.5 m wide and extending to a depth of 0.5 m); only snails of the sexually mature cohorts were sampled. Sampling was focused on gastropod snails belonging to the families Lymnaeidae and Planorbidae, since they proved to harbour the most diverse trematode fauna in Europe^42,46,58,59^. To standardise the sampling effort, sampling was carried out by three persons for 30 min at each site. Sampling times were extended at low densities of mature snails to obtain target sample size (see below). The sample sizes therefore provide an indirect measure of the abundance of the individual hosts at the sampling sites. In total, 3171 lymnaeid snails belonging to four species [1909 *Radix auricularia* (L.), 668 *Stagnicola palustris* (Müller), 349 *R. peregra* (Müller) and 245 *Lymnaea stagnalis* (L.)] and 2176 planorbid snails belonging to two species [1981 *Gyraulus albus* (Müller) and 195 *Segmentina nitida* (Müller)] were screened for trematode infections. Despite the debated taxonomic status of *R. peregra* (^60^ and references therein), we have kept our initial morphological identification of this host to be comparable with previous publications from this dataset^37,39^.

In the laboratory, all snails were placed in individual cups with filtered lake water and exposed to a light source for two to five days to induce cercarial emergence at 20 °C. Snails that did not emit cercariae during that time were dissected and screened for prepatent infections. Trematode stages were identified alive under an Olympus BX51 microscope with the help of appropriate identification keys or other relevant identification sources^61–64^ and documented with an Olympus UC30 digital camera. For further investigation of taxonomically problematic groups, trematode material was fixed in molecular biology grade ethanol and 4% formaldehyde solution for molecular and morphological studies, respectively, to reveal potential cryptic diversity. Voucher material for *Neopetasiger* spp. is deposited in the Collection of the Institute of Parasitology (Czech Academy of Sciences; accession numbers D707-D709 and photovouchers for *Diplostomum* spp. and *Echinostoma* sp. IG are included in^37,39^). Representative sequences were submitted to the GenBank database under the accession numbers KC618448-KC618461 (*Echinostoma* spp.); KR149504-KR149554 and KR149490-KR149503 (*Diplostomum* spp.); and KM191799-KM191817 (*Neopetasiger* spp.). Raw sampling data (snail parasite community composition in each individual sample) is provided in Supplementary Table S1.

### Data analysis

Parasite biodiversity was studied at different hierarchical levels, the trematode fauna in the individual lakes, the trematode fauna of each snail host species, and the trematode composition at the component community level. A component community is defined as all parasite species exploiting a host population at a given point in time (i.e., all parasite species found in one snail population at a sampling site during one sampling event)^65^. Parasite prevalence (P, in %) was calculated as the number of hosts infected with a given parasite species (n_inf_) divided by the total number of hosts examined (N): P = n_inf_/N * 100^65^. Focusing on the mode of infection of the first intermediate host and the transmission pathways to definitive hosts we recognise two trematode groupings further referred to as ‘transmission guilds’ or ‘guilds’ for simplicity: either the guild of parasites that use fish as second intermediate and fish-eating birds as definitive hosts (fish-eating bird guild), or the guild of parasites that utilise anseriform birds as definitive hosts (waterfowl guild).

In order to identify patterns and structures at the component community level, component community composition analyses, i.e., randomisation tests on similarity matrices (ANOSIM) based on Bray-Curtis index values and non-metric multi-dimensional scaling (MDS) ordination were performed with PRIMER v6^66^. We tested the effect of the factors ‘lake’, ‘season’, ‘year’ and ‘snail host’ on the component community composition against a null hypothesis of no significant differences for these factors (one-way ANOSIM). In the MDS plots, each data point (represented by a symbol) corresponds to a parasite component community for a snail host population. The more similar the parasite communities are to each other, the closer the respective symbols group together. Different characteristics of the component communities can be visualised (e.g., snail host or lake) in order to analyse the structure of trematode communities. To reduce the bias due to small sample size, only data from individual samples consisting of ≥ 14 snails were used in the component community analyses; samples consisting of fewer snails were excluded from these analyses. This sample size was chosen as it allowed to include most samples from snail hosts that often occurred at low densities. Since trematode infections in snails often show prevalences within the 5–10% range, we considered trematode species with a prevalence higher than 10% in at least one component community as dominant (see^35,67^) to identify the most prevalent parasites. Individual rarefaction curves of the trematode communities were calculated to compare diversity in the different lymnaeid and planorbid snail populations (see Supplementary Fig. S2).

Most component community data were available from *R. auricularia* (48 samples), allowing further statistical tests for temporal and spatial variation in this host-parasite system. Five samples from the lake Hennetalsperre were excluded, since they were available from one year only (2012) and no samples were obtained from spring. Accordingly, based on 43 component communities we tested the temporal and spatial variation for three lakes (Baldeneysee, Hengsteysee and Sorpetalsperre), i.e., detecting community structure by comparing communities between different lakes, seasons and years. For the factor ‘season,’ individual samples were grouped as follows: spring (May), summer (June – August) and autumn (September). First, we tested differences in sample size and snail size (mean snail length) in relation to factors ‘lake’, ‘season’ and ‘year’ by separate one-way analysis of variance (ANOVA). Secondly, we tested whether sample size was correlated with overall prevalence and species richness (Spearman’s rank order correlations). Mean snail size (snail shell length) in the individual *R. auricularia* component communities varied considerably (9.6–22.5 mm, mean length ± SD, 14.6 ± 3.5 mm). Therefore, we tested the possible relationship of mean snail size with overall prevalence and species richness (Spearman’s rank order correlations). If both analyses indicated significant differences for snail size related to the factors tested (ANOVA), and its positive correlation with overall prevalence, suggesting higher infection levels in communities in larger snails, we used more appropriate statistical test to evaluate the spatio-temporal effect on the overall prevalence by two-way analyses of covariance (ANCOVA), while controlling the effect of snail size (entered as a covariate). Prepatent infections, which were identified as sporocysts or rediae and could not be assigned to any trematode species due to their immature stage, were excluded from the analyses. Similarly, rare trematode species which occurred in less than three component communities were not included. This resulted in the 15 most common species (eight were excluded out of 23) which were entered into analyses testing the variation of prevalence in space and time. For these analyses we used two datasets: (i) for the analysis of variation between ‘lakes and seasons’ and ‘lakes and years’, 43 component communities from three lakes and three seasons (spring, summer and autumn) were included (ANCOVA 1); (ii) for the analysis of seasonal variation (’season and year’), six spring samples were excluded, since they were from one year (2012) only. Therefore 37 component communities from three lakes and two seasons (summer and autumn) were included into this analysis (ANCOVA 2). The same procedure was followed while analysing seasonal and temporal patterns in prevalence of the transmission guilds (six species in the waterfowl guild and five species in the fish-eating bird guild). Post-hoc Tukey HSD tests were performed where appropriate. To improve the fit of the normal distribution, data on sample size and snail size (mean length) were log_10_ (x) transformed, prevalence data (expressed as proportions) were arcsin square-root-transformed, and species richness data log_10_ (x + 1) transformed. All tests were carried out with Statistica v.7 (StatSoft Inc.).

## Supplementary information

Supplementary Table S1 Raw sampling data of trematode infections in lymnaeid and planorbid snails from the Ruhr area.

Supplementary Figure S2 Rarefaction curves of trematode communities found in lymnaeid and planorbid snail populations of the Ruhr area.

## Data Availability

Subsets of the data have already been published (e.g., concerning the cryptic species diversity in the system) and are referenced accordingly. Voucher material for *Neopetasiger* spp. is deposited in the Collection of the Institute of Parasitology (Czech Academy of Sciences) (accession numbers D707-D709 and photovouchers for *Diplostomum* spp. and *Echinostoma* sp. IG are included in^37,39^. Representative sequences were submitted to the GenBank database under the accession numbers KC618448-KC618461 (*Echinostoma* spp.); KR149504-KR149554 and KR149490-KR149503 (*Diplostomum* spp.); and KM191799-KM191817 (*Neopetasiger* spp.). All raw sampling data are available in Supplementary Table S1.
