# Supplementary material for: Hidden parasite diversity in a European freshwater system
Source: Sci Rep. 2020 Feb 14;10:2694. doi: 10.1038/s41598-020-59548-5 (PMC7021786; doi:10.1038/s41598-020-59548-5)

Supplementary Figure S2

Publication: Hidden parasite diversity in a European freshwater system

Authors: Christian Selbach, Miroslava Soldánová, Christian K. Feld; Aneta Kostadinova, Bernd Sures

Description: Figure showing individual rarefaction curves of the trematode communities found in the lymnaeid and planorbid snail populations of the Ruhr area, Germany, based on (A) trematode species richness and (B) Shannon H diversity indices.

All calculations performed with Past 4.0 (Hammer et al. 2001).

Reference: Hammer, Ø., Harper, D.A.T., and P. D. Ryan, 2001. PAST: Paleontological Statistics Software Package for Education and Data Analysis. Palaeontologia Electronica 4(1): 9pp.

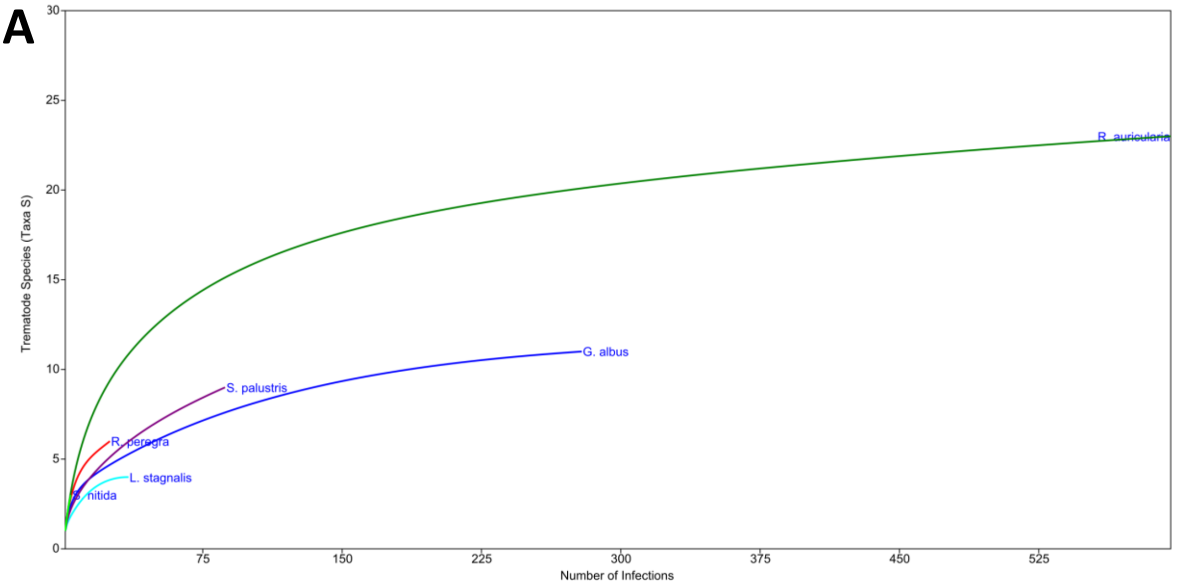

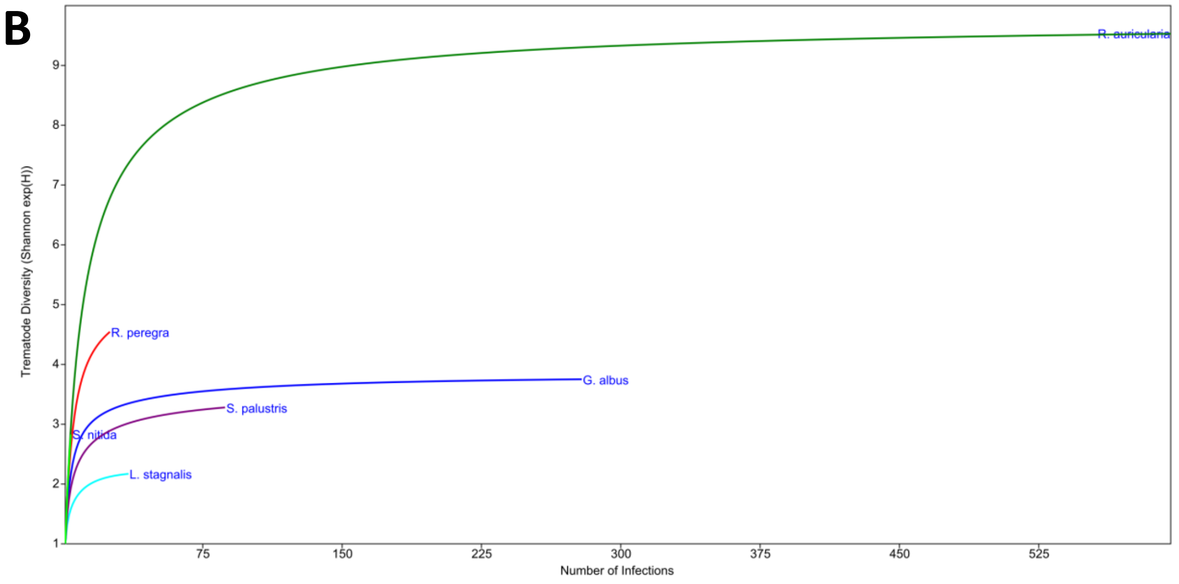

Supplement: Supplementary file 2 — Supplementary Figure S2 Rarefaction curves of trematode communities found in lymnaeid and planorbid snail populations of the Ruhr area. [file 41598_2020_59548_MOESM2_ESM.pdf]
